# Breast Reconstruction after Blunt Breast Trauma: Systematic Review and Case Report Using the Ribeiro Technique

**DOI:** 10.1055/a-2121-7560

**Published:** 2023-11-30

**Authors:** Horacio F. Mayer, René M. Palacios Huatuco, Mariano F. Ramírez, Ignacio T. Piedra Buena

**Affiliations:** 1 Plastic Surgery Department, Hospital Italiano de Buenos Aires, University of Buenos Aires Medical School, Hospital Italiano de Buenos Aires University Institute, Buenos Aires, Argentina

**Keywords:** blunt injury, breast reconstruction, traffic accident, seat belts

## Abstract

Blunt breast trauma occurs in 2% of blunt chest injuries. This study aimed to evaluate the evidence on breast reconstruction after blunt trauma associated with the use of a seat belt. Also, we describe the first case of breast reconstruction using the Ribeiro technique. In November 2022, a systematic search of MEDLINE, EMBASE, and Google Scholar databases was conducted. The literature was screened independently by two reviewers, and the data was extracted. Our search terms included breast, mammoplasty, blunt injury, and seat belts. In addition, we present the case of a woman with a left breast deformity and her reconstruction using the inferior Ribeiro flap technique. Six articles were included. All included studies were published between 2010 and 2021. The studies recruited seven patients. According to the Teo and Song classification, seven class 2b cases were reported. In five cases a breast reduction was performed in the deformed breast with different types of pedicles (three superomedial flaps, one lower flap, one superior flap). Only one case presented complications. The case here presented was a type 2b breast deformity in which the lower Ribeiro pedicle was used successfully without complications during follow-up. Until now there has been no consensus on reconstructive treatment due to the rarity of this entity. However, we must consider surgical treatment individually for each patient. We believe that the Ribeiro technique is a feasible and safe alternative in the treatment of posttraumatic breast deformities, offering very good long-term results.

## Introduction

Blunt breast trauma is a rare entity and occurs in 2% of blunt chest injuries. 1 Seat belt use has significantly reduced the number of deaths associated with motor vehicle accidents. 2 However, a new pattern of injuries has emerged called “seat belt syndrome,” which encompasses a broad spectrum of injuries, including soft tissue injuries to the breast. Traumatic breast injuries range from bruising to avulsion, caused by compression of the breast between the bony rib cage and the seat belt. 3 Repairing cosmetic sequelae from severe breast trauma represents an unusual challenge in breast reconstruction. The aim of this study was to present a systematic review of the literature on breast reconstruction after blunt trauma associated with the use of a seat belt. Also, we describe the first case of breast reconstruction using the Ribeiro technique.

## Case

**Fig. 1:**
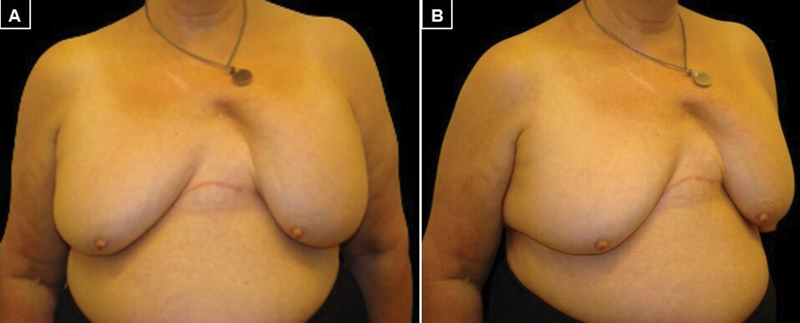
Preoperative images showing the medial defect in the left breast. ( **A** ) Front view. ( **B** ) Oblique view.

**Fig. 2:**
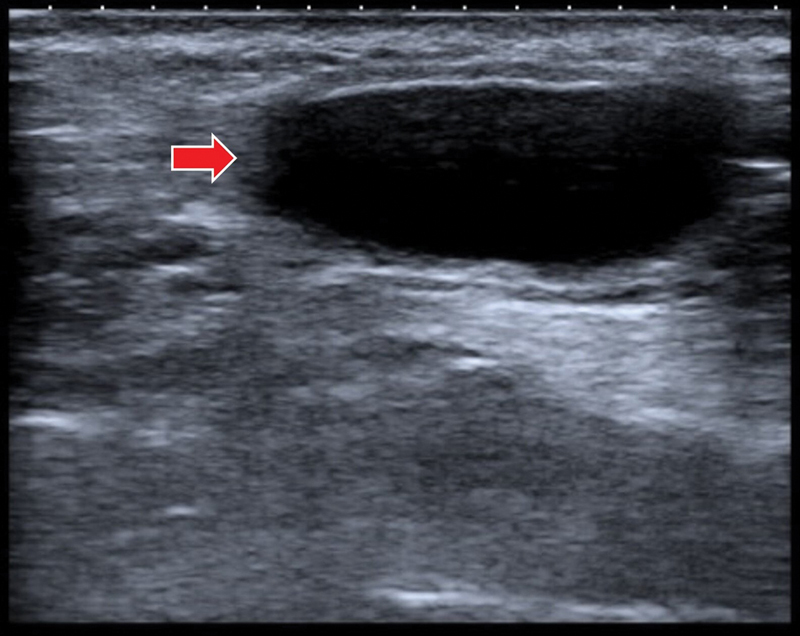
Breast ultrasound showing a 7 cm × 5 cm cystic image with thin walls (red arrow).

A 58-year-old woman with a history of severe chest trauma in a car accident, during which she was wearing a seat belt. She presented fractures of the right upper extremity, nose, and multiple rib fractures, for which she required an anterolateral thoracotomy approach and reduction and osteosynthesis of three right ribs, radius, and distal ulna. The patient was consulted 7 months later in our Plastic Surgery Department due to a deformity in the left breast. On physical examination, she presented third-degree bilateral breast ptosis and an inframammary scar extending from the right midaxillary line to the left sternal border. In addition, she had a marked asymmetry due to a large soft tissue defect in the superomedial pole of the left breast. This defect caused retraction of the breast tissue and medialization of nipple–areola complex (NAC; Fig. 1 ). Breast deformity was classified as class 2b of the Teo and Song classification ( Table 1 ). 4 Mammographic studies demonstrated a 7 × 5 cm cytosteatonecrosis cyst in the inferomedial quadrant of the left breast ( Fig. 2 ).

**Table 1:** Teo and Song classification

Class	Presentation
1a	Immediate presentation with bruising and pain of the breast with or without minor wounds
	New breast asymmetry for patients with implants in situ
1b	Immediate presentation with expanding breast
2a	Late presentation with tender breasts, mild bruising, or palpable lump
2b	Late presentation with structural distortion (breast cleft or capsule contracture)

### Surgical Technique

**Fig. 3:**
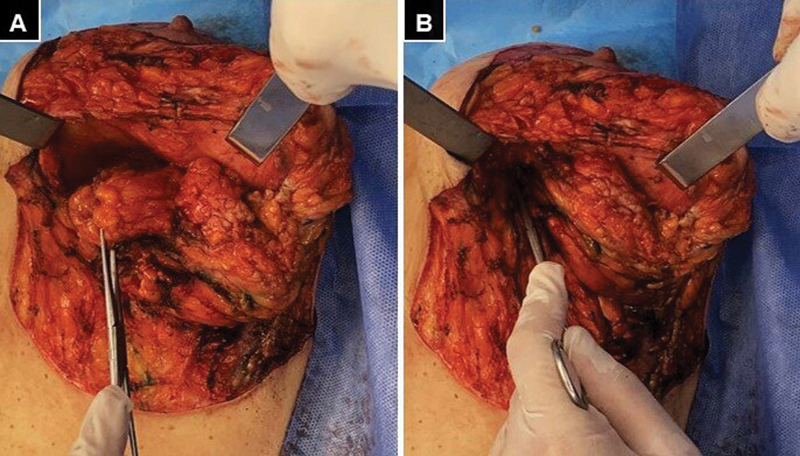
Intraoperative images of the left breast. ( **A** ) Preparation of the lower pedicle that should measure between 2 and 3 cm thick. ( **B** ) Fixation of the pedicle in the superomedial sector of the fascia of the pectoralis major muscle.

The preoperative marking was based on the Pitanguy technique for breast reduction, 5 and the procedure was performed under general anesthesia. A solution with 2% xylocaine and 1/100,000 dilution adrenaline was used to infiltrate the area. We performed epidermalization of the left breast, including resection of the anterolateral thoracotomy scar. The inferomedial quadrant was approached and a large cyst of cytosteatonecrosis was found that was resected. In addition, a scar fibrosis area was identified that corresponded to the soft tissue defect, which caused considerable loss of breast tissue. To cover the medial breast defect and improve its shape, we designed a lower flap 4 to 5 cm wide and 2 to 3 cm thick ( Fig. 3 ), according to the Ribeiro technique. 6

**Fig. 4:**
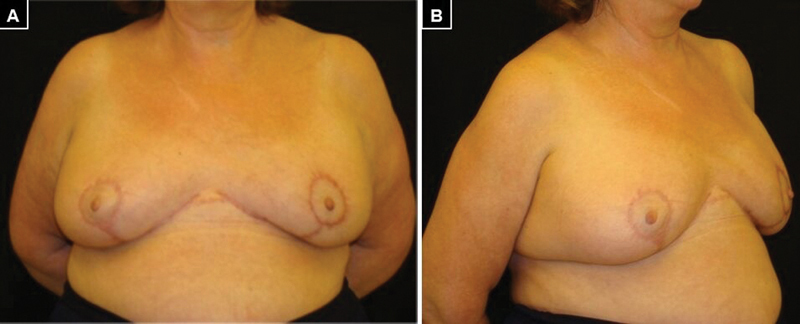
Postoperative images. ( **A** ) Front view. ( **B** ) Oblique view.

This flap was deepithelialized and an incision was made along its edges down to the muscular fascia, from which it was detached. The lower pedicle was maintained through which the fourth, fifth, and sixth intercostal perforators penetrated, thus ensuring irrigation of the dermo-lipo glandular flap. After de-epidermizing the pedicle and periareolar area previously designed to correct the location of the NAC, the dermoglandular flap was fixed to the pectoralis major at the level of the second left intercostal space in the superomedial pole. Finally, contralateral breast symmetrization was performed by reduction and mastopexy with inverted T. No complications were reported and hospital discharge was granted on the first postoperative day. The evolution was favorable during the 12-month follow-up, with excellent cosmetic results and patient satisfaction ( Fig. 4 ).

### Systematic Review of the Literature

This study followed the Preferred Reporting Items for Systematic Reviews and Meta-Analyses (PRISMA) reporting guidelines. 7 We performed a literature search from inception until November 2022 in the following databases: MEDLINE, EMBASE, and Google Scholar. For the initial search, the following keywords and MeSH terms were used: “Breast” AND “Mammoplasty OR Breast reconstruction” AND “Blunt injury OR Nonpenetrating Wound” AND “Traffic accident OR Seat Belts.”

### Study Selection

Initial screening of articles by title and abstract was performed independently by two reviewers, selecting relevant studies according to eligibility criteria. A third author resolved any inclusion conflict using Rayyan software. 8 The inclusion of articles was limited to (1) articles published from inception up to November 2022; (2) reports in English and French; (3) female patients over 18 years old; (4) patients with blunt breast trauma; (5) seat belt use; and (6) patients with cleft deformity or breast distortion in the line of the seat belt. Meanwhile, studies were excluded if they met one or more of the following criteria: (1) language other than English and French; (2) meta-analyses or systematic reviews; (3) male patients; (4) patients with breast trauma as a result of a different mechanism of injury; and (5) reports of conservative, minimally invasive treatment or studies that did not report surgical reconstruction of breast deformity.

### Data Extraction

The following information was extracted: first author's name, the publication year, and the country, patients' age and sex, the presentation of breast deformity, intraoperative findings, surgical reconstruction technique, complications, and follow-up results.

### Statistical Analysis

Meta-analysis could not be performed because this systematic review includes an extremely rare entity based on published cases. However, the extracted data from the cases were put on a sheet and then quantitatively analyzed using descriptive statistics.

**Fig. 5:**
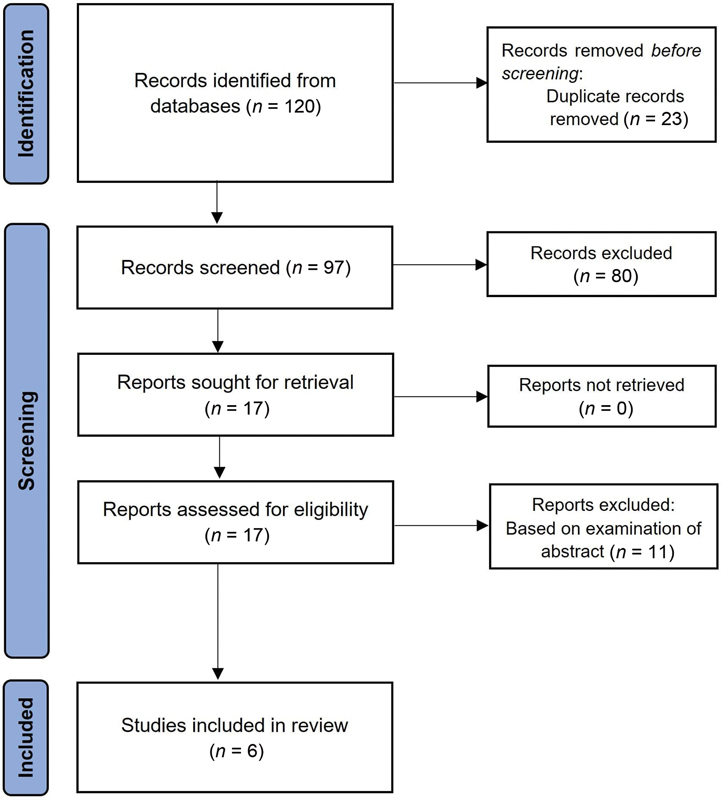
Preferred Reporting Items for Systematic Reviews and Meta-analyses flowchart of the study selection process.

The search resulted in 120 records, of which 23 were duplicates. After title and abstract screening, 91 articles were excluded. This systematic review included six published articles 3 4 9 10 11 12 after full-text assessment. The PRISMA flow diagram presents the study selection process ( Fig. 5 ).

### Study Characteristics

This systematic review of published cases included six articles with seven patients ( Table 2 ). The year of published case reports ranged from 2010 to 2021, of which 67% were published in the past 10 years. Most of the published cases are from the United Kingdom (three cases), followed by France (two cases). According to the Teo and Song classification, seven class 2b cases were reported. The mean age of the patients was 57.6 years (range: 37–60). In all cases, the affected breast was on the right side and a diagonal cleft predominated with NAC retraction. The most frequent intraoperative finding was fat necrosis, which could be accompanied by fibrosis. Regarding surgical techniques, in five cases a breast reduction was performed on the deformed breast with different types of pedicles (three superomedial flaps, one lower flap, one superior flap), and only in two cases was the breast reconstruction technique described in detail. One case presented complications (delayed wound healing of the inferior incisions). Median follow-up was 7.5 months (range: 3–12) with satisfactory cosmetic results.

**Table 2:** List of cases of women with breast reconstruction after blunt trauma with a seat belt

No.	Study (year)	Country	Age	Presentation	Intraoperative findings	Reconstruction technique	Complication	Follow-up (mo)
1	Paddle and Morrison (2010) 3	Australia	37	Right diagonal cleft + NAC retraction	Fat necrosis	Right mastopexy + left reduction	No	6
2	Scevola et al(2011) 9	France	60	Right diagonal cleft + NAC retraction	Fat necrosis	Mastopexy + bilateral reduction (superomedial flap) + lipofilling	No	12
3			54	Right diagonal cleft	Fat necrosis		No	12
4	Teo et al (2014) 16	The United Kingdom	67	Right diagonal cleft + NAC retraction	Fat necrosis	Resection + deepithelialization of the edges of the cleft	No	2
5	Petrie (2014) 10	The United Kingdom	69	Right diagonal cleft + NAC retraction	Fat necrosis	Breast reduction (superomedial flap) + lipofilling	No	–
6	Noel et al (2020) 11	The United States	53	Right diagonal cleft	Fat necrosis + fibrosis	Bilateral reduction (lower flap)	Delayed wound healing of inferior incisions	4
7	Lafford et al (2021) 12	The United Kingdom	63	Right vertical cleft + NAC inversion	Fibrosis	Bilateral reduction (superior flap)	No	3
8	Our case	Argentina	58	Left soft tissue defect + NAC retraction	Fat necrosis + fibrosis	Mastopexy + bilateral reduction (lower flap type I Ribeiro)	No	12

Abbreviation: NAC, nipple–areola complex.

## Discussion

Mechanisms of seat belt breast trauma include both shearing and crushing injuries resulting from shoulder restraint. Most patients have associated injuries that, due to their severity, require immediate treatment. The most common are long bone extremity fractures (47%), rib fractures (15%), solid organ injury (11%), and pneumothorax or hemothorax (10%). 1 This coincides with the associated lesions in our patient.

Two classification systems have been proposed for blunt breast trauma with the aim of stratifying the injuries and establishing adequate treatment. In 2007, the first classification of seat belt injuries in the female breast was described, categorized according to the type and severity of the injury at the time of the traffic accident. 13 However, it did not include late presentations, pregnant patients, or patients with breast prostheses. Subsequently, in a systematic review, a four-level classification was presented that categorizes patients according to the time of presentation of symptoms and their treatment, representing the most complete report that summarizes all published literature on seat belt-associated breast injuries. 4

Our case corresponds to class 2b, which includes severe aesthetic sequelae and a late presentation with cleft deformity in the seat belt line or breast distortion, with reconstruction being the main treatment. Class 2b breast deformity represents a significant reconstructive challenge in restoring the natural contour of the breast, the inframammary fold, and repositioning of the NAC. 12 So far, seven class 2b cases have been reported discussing different reconstruction options in seat belt-associated breast trauma. In all cases, the affected breast was on the right side and a diagonal cleft predominated with NAC retraction. In contrast, our patient presented involvement of the left breast with medialization of the NAC. This type of breast deformity was present late in breasts with certain anatomical characteristics such as the presence of a greater volume in the lower pole with a predominance of adipose tissue, lax skin, and third-degree ptosis. We estimate that skin retraction and NAC occurred as a result of fat necrosis and fibrosis, which were found to be the most frequent intraoperative findings. This presentation also coincides with our case.

On the other hand, this breast morbidity has not been described in patients with breast implants, 14 15 probably because implants interposed as a damping mechanism between the bony rib cage and soft tissues when seat belts dynamically restrain people in their seats.

Regarding reconstruction techniques, they were individualized for each patient according to the type of breast deformity. However, the surgical techniques used were described in detail only in two cases. Paddle and Morrison 3 described a modified Hall–Findlay-type mastopexy with good cosmetic results. Similarly, Teo et al 16 reported an approach in which they resected and deepithelialized the edges of the cleft, with subsequent elevation of the NAC in a superolateral pedicle while creating and mobilizing the superomedial and inferolateral crura to close the oblique breast defect. Furthermore, Lafford et al 12 performed NAC retraction release with resection of the edges of the defect and subsequent bilateral breast reduction with a superior flap. On the other hand, Petrie 10 resected the edges of the defect but limited the incision from the lower edge of the cleft to a point on the upper edge of the NAC to avoid leaving a visible scar in the cleavage area of the breast. Scevola et al 9 applied conventional breast reduction techniques and a posterior glandular flap in gland reshaping and lipofilling to fill in substance loss and improve breast contour. Noel et al 11 designed a modified inferior dermoglandular pedicle to correct the defect and standard reduction in the contralateral breast. However, they do not describe the surgical technique used.

Breast reduction techniques would appear to be the best indication to correct the defect generated by blunt trauma in ptotic breasts. Therefore, the specific reduction technique, as well as the different pedicle techniques selected, must consider the physical characteristics of the patient and the surgeon's experience. On the other hand, the inferior pedicle technique is currently a popular approach for breast reduction. This technique is useful for the correction of breast asymmetries and ptosis and can be used on virtually any breast size and shape with a high degree of patient satisfaction. 17

In contrast to what has been reported so far, we describe reconstruction of the breast defect using the Ribeiro inferior glandular dermo-lipo flap technique. 6 This flap was presented for the first time in Brazil at the Congress of the Brazilian Society of Plastic Surgery in 1971. This technique, also known as the “first pedicle or safety pedicle,” was designed to provide safety in terms of volume in the case of reductions, greater projection of the upper pole, and more lasting results in the cases of mastopexies. 18 During the past 30 years, the technique has been adapted to allow the development of five different types of flaps with different indications. 6 Due to its versatility and use, not only in cosmetic surgery but also in reconstructive surgery, we can consider it an extraordinary resource in breast plastic surgery.

In relation to breast deformities in blunt trauma, we can find a wide spectrum of presentations; therefore, there is no adopted reconstruction standard. However, we must consider surgical treatment individually for each patient.

Blunt mammary trauma associated with seat belt use is rare; therefore, reconstructive treatment implies a challenge for the plastic surgeon, who must evaluate each case individually and establish the most appropriate surgical strategy. This is the first report to describe breast reconstruction with the Ribeiro inferior flap technique in reconstruction after blunt breast trauma associated with the use of seat belts. We believe that the Ribeiro technique is a feasible and safe alternative in the treatment of posttraumatic breast deformities, offering very good long-term results.
